# Emerging microbiota during cold storage and temperature abuse of ready-to-eat salad​

**DOI:** 10.1080/20008686.2017.1328963

**Published:** 2017-06-06

**Authors:** Karin Söderqvist, Omneya Ahmed Osman, Cecilia Wolff, Stefan Bertilsson, Ivar Vågsholm, Sofia Boqvist

**Affiliations:** ^a^ Department of Biomedical Sciences and Veterinary Public Health, Swedish University of Agricultural Sciences, Uppsala, Sweden​; ^b^ Department of Ecology and Genetics, Limnology, and Science for Life Laboratory, Uppsala University, Uppsala, Sweden

**Keywords:** Baby spinach, *Escherichia coli* O157:H7, *Listeria monocytogenes*, mixed-ingredient salad, pathogenic *Yersinia enterocolitica*, phyllosphere

## Abstract

**Introduction**: Ready-to-eat (RTE) leafy vegetables have a natural leaf microbiota that changes during different processing and handling steps from farm to fork. The objectives of this study were (i) to compare the microbiota of RTE baby spinach and mixed-ingredient salad before and after seven days of storage at 8°C or 15°C; (ii) to explore associations between bacterial communities and the foodborne pathogens *Listeria monocytogenes*, pathogenic *Yersinia enterocolitica*, and pathogen model organism *Escherichia coli* O157:H7 *gfp*+ when experimentally inoculated into the salads before storage; and (iii) to investigate if bacterial pathogens may be detected in the 16S rRNA amplicon dataset.

**Material and methods**: The microbiota was studied by means of Illumina 16S rRNA amplicon sequencing. Subsets of samples were inoculated with low numbers (50–100 CFU g^−1^) of *E. coli* O157:H7 *gfp*+, pathogenic *Y*. *enterocolitica* or *L*. *monocytogenes* before storage.

**Results and discussion**: The composition of bacterial communities changed during storage of RTE baby spinach and mixed-ingredient salad, with Pseudomonadales as the most abundant order across all samples. Although pathogens were present at high viable counts in some samples, they were only detected in the community-wide dataset in samples where they represented approximately 10% of total viable counts. Positive correlations were identified between viable counts of inoculated strains and the abundance of Lactobacillales, Enterobacteriales, and Bacillales, pointing to positive interactions or similar environmental driver variables that may make it feasible to use such bacterial lineages as indicators of microbial health hazards in leafy vegetables. The data from this study contribute to a better understanding of the bacteria present in RTE salads and may help when developing new types of biocontrol agents.​

## Introduction

Consumption of healthy and convenient foods such as ready-to-eat (RTE) leafy vegetables or mixed-ingredient salads has increased during recent years in many high-income countries. There has also been a concomitant increase in the number of reported foodborne disease outbreaks associated with leafy vegetables.[1,2]

Raw leafy vegetables host high numbers of naturally occurring microorganisms on the leaf surface (the phyllosphere), with bacteria as the most numerous colonists, reaching up to 10^8^ CFU g^−^^1.^[3] The leaf microbiota may be altered by various inputs of contaminants in the field environment or during subsequent processing and storage.[4] Microorganisms can also be found within plants as endophytic bacteria originating from soils surrounding the plant roots or from leaf surfaces, that then enter the host through the root system or via stomata or wounds because of mechanical damage.[5] Most microorganisms associated with leafy vegetables are considered harmless for humans, but human pathogens may occasionally occur as the result of exposure to contaminated irrigation water or manure, cross-contamination via animals, dirty equipment or human handling.[6] During processing, e.g. washing or cutting, the natural protective barriers of leafy vegetable cells are damaged and the intracellular nutrients they release may further enhance bacterial growth.[7] Storage conditions may also further influence the resident microflora.[8] Leafy vegetables and other food products containing this ingredient (e.g. mixed-ingredient salads), critically depend on good hygiene practices during production and processing, appropriate washing and a controlled cool chain to control microbial risks. There is however no step that effectively eliminates pathogens during the production of RTE leafy vegetables. Washing steps merely reduce the microbial load with 90–99% [6] and is unable to eliminate potential internalized pathogens.[9] The final product that is consumed will thus contain a large number of viable microorganisms, ranging from 10^7^ to 10^9^ viable cells per gram,[10,11] while occasionally also featuring foodborne pathogens.

Earlier work has shown that leafy vegetables host a diverse mixture of bacteria, yeasts, and fungi.[12] Many of the indigenous bacteria on leafy vegetables appear to be Gram-negative, including members of the Pseudomonadaceae and Enterobacteriaceae families.[13,14] Some bacterial strains isolated from leafy vegetables, including isolates of *Pseudomonas*, *Enterobacter*, and *Pantoea* spp., have shown an inhibitory effect against a range of pathogens under laboratory conditions.[15,16] It has been suggested that intentional addition of such antagonistic strains that are able to cope with the cool storage conditions typically used for commercial RTE salad products could serve as biocontrol agents to reduce the risk of pathogens colonizing leafy vegetables.[15,17] However the application by food business operators has been limited [18] and the efficiency of such procedures has yet to be demonstrated.

Food microbiological analysis has traditionally been based on culture-dependent approaches, with cultivation-independent molecular methods only recently introduced.[19] DNA-based methods enable detection of a broader range of bacteria that may have escaped detection by traditional cultivation. The culturable subset do typically not exceed 1% of the total community,[20] and moving microbial surveillance methods beyond this bottleneck now enables assessment and monitoring of complex bacterial communities in different food products. So far, few studies investigating microbiota in leafy vegetables have had a focus on the final product to be consumed, such as RTE leafy vegetables subject to refrigerated storage.[4]

The objective of the present study was to compare the bacterial communities before and after seven days storage of RTE baby spinach and mixed-ingredient salad (baby spinach mixed with chicken meat), at refrigerator temperature (8°C in Sweden) or temperature abuse conditions (15°C). Other aims were to explore associations between microbiota and the foodborne pathogens *L. monocytogenes*, *Y. enterocolitica*, and pathogen model organism *E. coli* O157:H7 *gfp+* when experimentally inoculated into the salads before storage and to investigate if these inoculants could be detected in the 16S rRNA amplicon dataset.

## Materials and methods

### Study design of growth trials from which salad materials originated

Samples for amplicon sequencing were collected at different time points during inoculum trials as described in detail in Söderqvist et al. [21]. Bags with RTE baby spinach (*Spinacea oleracea* L.) from the same production batch were used within each of the three trials. The baby spinach originated from Spain but was washed with potable water and packed at a Swedish processing plant. In samples with mixed-ingredient salad, commercial grilled diced chicken meat was added to the baby spinach. In brief, trials were set up as three-factorial studies with repeated measures. The fixed factors were (i) inoculant (*L. monocytogenes*, pathogenic *Y. enterocolitica*, *E. coli* O157:H7 *gfp+*, and un-inoculated control); (ii) addition of chicken meat (with or without); and (iii) storage temperature (8 ± 1°C or 15 ± 1°C). In total, 54 samples were collected for 16S rRNA amplicon sequencing during three trials. During each trial, one replicate of baby spinach and mixed-ingredient salad sample (controls), respectively, was collected on day 0. On day 7, this was repeated for both control samples and inoculated samples that had been stored at 8 or 15°C.

### Inoculated bacterial strains

To test for pathogen growth, strains of *L. monocytogenes* SLV-444 (CCUG 69007), *Y. enterocolitica* SLV-408 (CCUG 45643), and *E. coli* O157:H7 (shigatoxin 1 and 2 negative, eae positive, obtained from the Swedish public health agency, Solna Sweden (registry no. E81186)) were used in this study. The *L. monocytogenes* and *Y. enterocolitica* strains were resistant to rifampicin (200 µg ml^–1^), and the *E. coli* O157:H7 strain was resistant to ampicillin (100 μg ml^–1^), and labeled with green fluorescent protein (*gfp* *+*).[22] These strains were inoculated into samples at an initial level of 50–100 CFU g^−1^ with separate samples used for each inoculated strain.

### Sample collection and processing

At the start of the experiment (day 0) and after seven days of incubation at 8 and 15°C, the contents of individual bags of baby spinach or mixed-ingredient salad were homogenized as described in Söderqvist et al. [21]. The homogenate was subsequently used for total counts of aerobic viable bacteria and counts of inoculated strains as described in Söderqvist et al. [21]. A 10 ml-portion of homogenate was stored frozen at −70°C until extraction for DNA-based amplicon analysis.

Microbial DNA was extracted from 500 µl homogenate, using the Power soil DNA isolation Kit (MO BIO Laboratories Inc.,​ Carlsbad, CA, USA) according to the manufacturer’s instruction. The quality of the extracted DNA was assessed by gel electrophoresis (1% agarose) as previously described.[23] Parallel processing of negative controls did not generate any PCR products.

### 16S rRNA amplification and Illumina sequencing

Bacterial 16S rRNA genes were amplified using non-barcoded PCR primers covering the V3 and V4 region of the 16S rRNA gene; Bakt_341F (CCTACGGGNGGCWGCAG) and Bakt_805R (GACTACHVGGGTATCTAATCC). An initial amplification for 20 cycles was followed by 100-fold dilution of the resulting PCR product. After purification, the PCR products from this first PCR reaction were then tagged with 50 forward and reverse barcoded primers (7 bp) in a second PCR step as detailed by Sinclair et al. [24]. Two pools of 50 barcoded samples were prepared and sent to the SciLifeLab SNP/SEQ sequencing facility at Uppsala University for library preparation by TruSeq Sample Preparation Kit V2 protocol (EUC 15026486 Rev C, Illumina).

All PCR reactions were done in 20 µl reaction volumes using 1.0 U Q5 high fidelity DNA polymerase (NEB, UK), 0.25 µM primers, 200 µM of each dNTP and 0.4 µg bovine serum albumin (NEB, ​Ipswich, MA, USA). The thermal program consisted of an initial 95°C denaturation step for 5 min, a cycling program of 95°C for 40 s, 53°C for 40 s, 72°C for 60 s, and a final elongation step at 72°C for 7 min.

The concentration of resulting PCR product was estimated by Gel Pro analyzer 3.1 using a 100 bp DNA ladder.[23] Amplicons from individual samples were pooled in equal amounts. The pooled amplicons were purified by Qiagen PCR purification kit (Qiagen, Hilden, ​Germany) and quantified using the PicoGreen kit (Invitrogen,  Carlsbad, CA, USA). ​Subsequent Illumina sequencing was performed by the SNP/SEQ SciLifeLab facility hosted by Uppsala University (Sweden) using MiSeq, paired-end 300 bp chemistry.

### Data processing

Data processing including barcode demultiplexing, assembly, quality control, and removal of chimeric sequences was carried out using the Illumitag pipeline described in Sinclair et al. [24]. The clustering of the processed data was performed using the standard Mothur procedure for Illumina Miseq data.[25] Bacterial sequences were grouped into operational taxonomic units (OTUs) with a 97% sequence identity cut-off value and were taxonomically annotated by comparison of representative OTU sequences to the ribosomal database project (RDP, http://rdp.cme.msu.edu). Raw sequence data have been deposited to the NCBI sequence read archive under accession number SRP071791. Samples were normalized in R [26] by randomized subsampling to 1000 reads. Three samples with particularly low read counts (< 1000) were excluded from the normalization and subsequent analyses. Finally, OTUs represented by less than 10 reads were removed to avoid potential biases from sequencing errors and singletons.

### 16S rRNA sequencing of inoculated strains

DNA was extracted from each of the three inoculated strains (*L. monocytogenes*, *Y. enterocolitica*, and *E. coli* O157:H7 *gfp*+) using the Power soil DNA isolation kit as described above. The 16S rRNA gene was amplified using the same non-barcoded primers as for the community analyses. PCR conditions were the same but 1U Taq polymerase was used instead of Q5 high fidelity DNA polymerase. PCR products were purified using the QIAquick PCR Purification Kit according to manufacturer instructions (Qiagen). The Big Dye Terminator v3.1 (Applied Biosystems, Paisley, UK) was used for sequencing reactions and products were subsequently analyzed by capillary electrophoresis on the ABI3730XL DNA Analyzer (Applied Biosystems) at the Uppsala Genome Center. Each sequence was checked by sequence scanner version 1.0 software and the identity of each strain was confirmed by blastn (http://blast.ncbi.nlm.nih.gov/Blast.cgi).

### Changes in microbiota during storage

To explore changes in microbiota during storage, the average proportion and associated standard error for each bacterial order was calculated for non-inoculated baby spinach and mixed-ingredient salad on day 0 and after storage for seven days at 8 or 15°C. All statistical analyses in this study were performed in R versions 3.2.1 or 3.2.2.[26]

### Associations between growth of inoculated strains and microbiota composition

Spearman rank sum correlations were estimated between viable counts of each inoculated strain and changes in sample microbiota from day 0 and day 7. Since the viable counts of inoculated bacterial strains were negligible on day 0 (50–100 CFU g^−1^) the difference in counts between day 0 and day 7 for each sample was represented by absolute viable counts on day 7. The microbiota was evaluated at bacterial order level for each sample with comparisons made between proportions of different orders on day 0 and day 7.

To predict the correlation between abundances of different orders (X) and viable inoculate counts (Y), partial least square (PLS) modelling [27] was performed in SIMCA 12.0 software (Umetrics AB, Umeå, Sweden). To identify X-variables which had the highest influence on the PLS model, variable importance in projection (VIP) scores were evaluated and X-variables with VIP >1 were regarded as the most important. The predictability power (Q^2^cum) which explains the proportions of variance in viable inoculate counts that was explained by the two main PLS-components was evaluated for all models. The probability of model prediction was tested by performing a 100 permutation model validation for every Y-variable. Skewed variables were log-transformed to obtain normal distribution prior to the analyses.

To evaluate if there was any association between the change in microbiota from day 0 to day 7, and any of the inoculated strains, a Kruskal–Wallis test was conducted for each of the 15 order levels with the type of inoculated strain (*L. monocytogenes*, *Y. enterocolitica* or *E. coli* O157:H7 *gfp*+ or control samples) as factors.

## Results

### Bacterial diversity and community composition

At the highest taxonomic resolution, between 18 and 90 OTUs (average 58 OTUs) were detected in each sample. For each sample with plain baby spinach, an average of 66 OTUs was detected, while the corresponding number for samples with mixed-ingredient salad was 50 OTUs. Across all samples, a total of 190 OTUs were detected, representing four different bacterial phyla. Relative abundances of bacterial phyla associated with control samples of baby spinach and mixed-ingredient salad are shown in Supplementary Table 1.

**Table 1.** Spearman rank sum correlation coefficients for the order levels showing significant (*p* ≤ 0.05) correlations to viable counts of inoculated strains day 7.

	Bacillales	Enterobacteriales	Lactobacillales
*L. monocytogenes* (*n *= 12)	0.88 (*p *< 0.001)	0.78 (*p *= 0.003)	0.84 (*p *< 0.001)
*Y. enterocolitica* (*n *= 12)	0.72 (*p *= 0.008)	0.85 (*p *< 0.001)	0.79 (*p *= 0.002)
*E. coli* O157:H7 *gfp+* (*n *= 10)	0.45 (*p *= 0.19)	0.20 (*p *= 0.58)	0.64 (*p *= 0.046)

**Figure 1.**
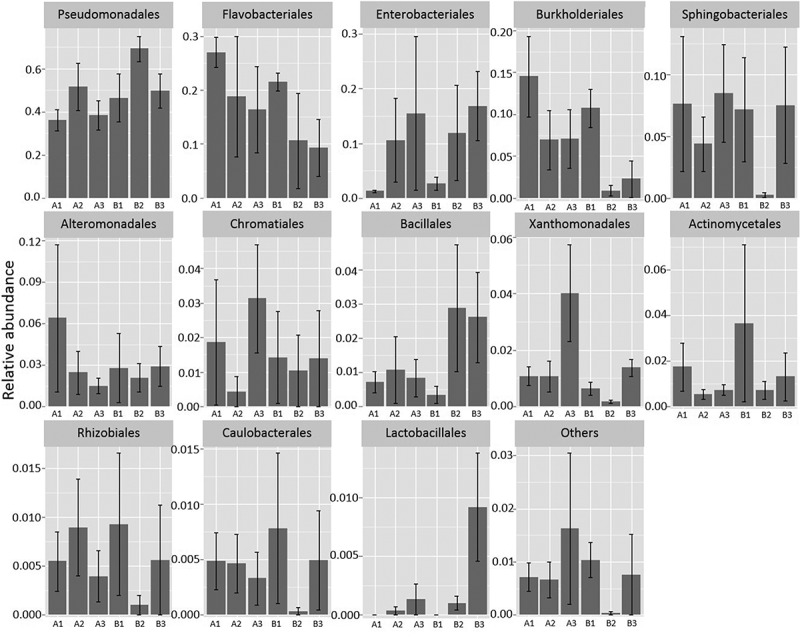
Relative abundance of bacterial orders in control samples of baby spinach and mixed-ingredient salad before and after storage at 8 and 15°C. A1 = baby spinach, day 0; A2 = baby spinach, day 7, stored at 8°C; A3 = baby spinach, day 7, stored at 15°C; B1 = mixed-ingredient salad, day 0; B2 = mixed-ingredient salad, day 7, stored at 8°C; B3 = mixed-ingredient salad, day 7, stored at 15°C. Note that Y-axes have different scales. The vertical bars represent standard errors.​

​  Pseudomonadales was the dominant order on day 0 and day 7, both for samples with plain baby spinach and mixed-ingredient salad and regardless of storage temperature. The highest proportion of this group in mixed-ingredient salad was observed after seven days at 8°C (approx. 70%; Figure 1). Flavobacteriales was the second most prevalent order in samples before storage (day 0), but their relative abundance decreased during storage at both temperatures and a similar decline was observed for Burkholderiales. In contrast, the proportion of Enterobacteriales was initially small (<5%) but increased to approximately 10–20% of the total community after seven days of incubation. For some bacterial orders, there were differences in their contribution to the community between samples of plain baby spinach and mixed-ingredient salad (Figure 1). Bacteria affiliated with Bacillales were for example present in very low proportions on day 0, but increased markedly during storage at 8 and 15°C in mixed-ingredient salad, while no such growth was observed in plain baby spinach. Lactobacillales were not detected in the beginning of the experiment (0 reads), but increased to approximately 1% of the total community in mixed-ingredient salad on day 7 when stored at 15°C. No such increase was observed in plain baby spinach. The order Lactobacillales contain many families, however only Carnobacteriaceae (genus *Carnobacterium*) and Enterococcaceae (genus *Vagococcus*) were detected in our study.

Robust identification at the species level was not possible given the incomplete 16S rRNA sequence (approximately 425–430 bp), but identification to the level of genus was in general feasible. At this level, the genus *Pseudomonas* was the most represented, with the highest relative abundance in samples stored at 8°C for seven days (mean 50 and 66% of reads in baby spinach and mixed-ingredient salad samples, respectively). Other important genera were *Acinetobacter*, *Flavobacterium*, *Erwinia*, *Spingobacterium*, *Shewanella*, *Stenotrophomonas*, *Duganella*, *Chryseobacterium*, and *Psychrobacter*. Some OTUs could not be classified at genus level but were instead classified as *Enterobacteriaceae* at family level. These represented 1–7% of the reads present in the sample types, with the highest proportion in mixed-ingredient salad, stored at 15°C.

For most of the samples in this study the number of reads that were lost during the bioinformatics analysis was half or less than half of reads initially generated (Supplementary Table 2). The loss of reads was mainly due to the presence of assembled and unassembled pairs with mismatched barcodes, sequences with low quality scores and sequence errors in different primer regions, and was of similar magnitude as previously described in Sinclair et al. [24].

### Associations between growth of inoculated strains and resident microbiota

**Figure 2.**
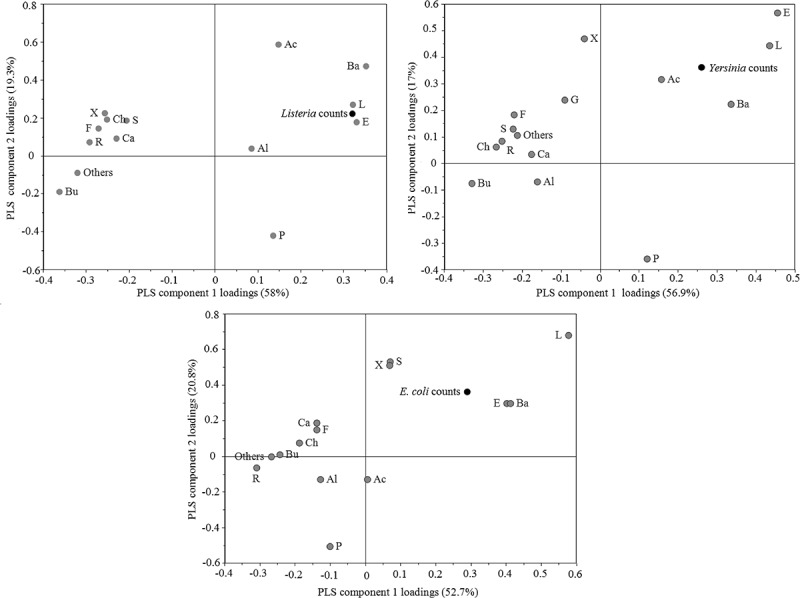
Loadings of the PLS regression analysis of order taxonomical composition prediction of viable counts of inoculated strains. The graph shows how Y-variables representing viable counts of (a) *Listeria monocytogenes*, (b) pathogenic *Yersinia enterocolitica* and (c) *Escherichia coli* O157:H7 *gfp*+ correlate with X-variables representing orders as follows: P, Pseudomonadales; F, Flavobacteriales; E, Enterobacteriales; Bu, Burkholderiales; S, Sphingobacteriales; Al, Alteromonadales; Ba, Bacillales; X, Xanthomonadales; Ac, Actinomycetales; Ch, Chromatiales; L, Lactobacillales; R, Rhizobiales; Ca, Caulobacterales; G, Unclassified gammaproteobacteria ​and Others. The plot can be read by drawing a line from the Y-variable through the origin and across the plot. X-variables situated near Y-variables are positively correlated to them and those situated on the opposite side are negatively correlated. The X-variables situated near each other are correlated.​

Viable counts of all inoculated strains (*L. monocytogenes*, *Y. enterocolitica*, and *E. coli* O157:H7 *gfp+*) after seven days of incubation were positively correlated to an increase in relative abundance of Lactobacillales in the microbiota, with Spearman rank correlation coefficients given in Table 1. Viable counts of *L. monocytogenes* and pathogenic *Y. enterocolitica* after seven days of incubation were also positively correlated to increasing relative abundances of Enterobacteriales and Bacillales (Table 1). These results were in large consistent with the observed positive correlations of viable counts of inoculated strains with the orders Bacillales, Lactobacillales, and Enterobacteriales in PLS loading plots (Figure 2(a–c)). These orders were also among the most influential in explaining the viable counts of *L. monocytogenes*, *Y. enterocolitica*, and *E. coli* O157:H7 *gfp*+ counts based on VIP scores. Rhizobiales, Burkholderiales, and Flavobacteriales were negatively correlated to all inoculate counts (Figure 2(a–c)) while the representation of Pseudomonadales in the community was only negatively correlated to *E. coli* O157:H7 *gfp*+ counts (Figure 2(c)). The power of prediction (Q^2^cum) was high for all models: 0.82, 0.55, and 0.61 for *L. monocytogenes*, pathogenic *Y. enterocolitica*, and *E. coli* O157:H7 *gfp+*, respectively.

The type of inoculated strain (or absence thereof in the controls) did not appear to affect the composition of the bacterial orders in the microbiota, according to Kruskal–Wallis test (*p* ≥ 0.58).

### Comparing sequences and culture counts of inoculated strains with OTUs

The mixed-ingredient salads stored at 15°C for seven days featured the highest absolute viable counts of inoculated strains with *L. monocytogenes* concentrations of approximately 10^9^ CFU g^−1^ and this was paralleled by abundance distribution patterns for one highly correlated OTU. This OTU was affiliated with *Listeria* and was >99% identical to the 16S rRNA sequence from the inoculated *L. monocytogenes* strain (Supplementary Table 3). The mean representation of this OTU in the total community was 2.5% for these samples. There was no OTU classified as *Escherichia/Shigella* in the normalized OTU table; however, one OTU with this classification was found when studying OTUs with lower reads than those included in the normalized dataset used for statistical tests. The sequences representing this particular OTU were > 97% identical to the 16S rRNA sequence from the inoculated *E. coli* O157:H7 *gfp*+ strain (Supplementary Table 3) but could still represent the same population as OTUs represented by a single sequence were clustered at 97% identity level. There were low numbers of reads for this OTU (between 3 and 22) in the three salad samples with the highest viable counts of the inoculated strain (approximately 10^7^ CFU g^−1^), while the number of reads were even lower for the remainder of the samples (≤ 1). The mean representation of this OTU in the total community was 0.2% for the samples with the highest concentration of *E. coli* O157:H7 *gfp*+, thus falling below the limit of detection after sequencing depth normalization. We were not able to correlate any OTU with the inoculated *Y. enterocolitica* strain, despite high viable counts of this strain in some samples (approximately 10^7^ CFU g^−1^). The OTUs most closely matching the 16S rRNA sequence of the *Yersinia* strain (Supplementary Table 3) were less than 97% identical and were classified as the family Enterobacteriaceae at the most highly resolved taxonomic level (unclassified at genus level).

## Discussion

The number of OTUs in each sample in this study (average 58) can be interpreted as the approximate number of bacterial species that a consumer likely will be exposed to when consuming RTE salad. The number of OTUs for plain baby spinach (average 66) is within the range of 20–80 OTUs that has previously been reported for RTE spinach.[5,28] The lower number of OTUs for mixed-ingredient salad (average 50) may at first appear surprising as there are more nutrients available in these samples compared to the plain baby spinach. However, such an increase in nutrient availability may promote rapid growth of opportunistic bacterial populations that would then mask the presence of the spinach-associated microflora. This is in line with earlier studies of diversity–productivity relationships where increased resource availability was sometimes seen to cause a decrease in diversity for exactly this reason.[29]

The rather low diversity that was observed in this study, based on number of phyla (*n* = 4), may be because the baby spinach used in our study had already been processed (i.e. washed in potable water), packaged and transported from the packaging facility to the retail store before being analyzed during the trials. Earlier work has shown that bacterial diversity in the phyllosphere of leafy vegetable samples collected from the field is greater than the diversity after packaging and storage.[8,30] For example, storage at 4°C during one day reduced the number of phyla from 11 to 5.[8]

In this study, results were based on one replicate sample from each of the three experiments performed. Increased number of replicates per sample category (plain baby spinach or mixed-ingredient with or without an inoculated strain) would have made the analyses and inferences more robust, both regarding viable counts and relative abundance of different OTUs, and should preferably be incorporated in future studies.

In the present study, most OTUs were classified at their genus level while species identity was not possible. In addition, few genera were not robustly separated and statistical analyses were therefore performed at order level. Most orders were represented by mainly one genus, for example Pseudomonadales that mainly featured *Pseudomonas*. However, a few orders contained several genera with different temporal dynamics, for example Enterobacteriales that was represented by *Erwinia* and unclassified Enterobacteriaceae. Still, we were able to reveal major changes in the microbiota based on the order level.

We observed that Pseudomonadales (mostly represented by genus *Pseudomonas*) was the order with the highest relative abundances in the resident microbiota. This is in accordance with results from other studies that have shown that *Pseudomonas* spp. were among the dominant bacterial populations in RTE spinach and mixed vegetable salads during cool storage.[8,14,31] *Pseudomonas* spp. are widely distributed in the environment and some species are potential plant pathogens [32] or pectinolytic species important for spoilage of vegetables.[33] It has been shown that when screening bacterial isolates naturally present on leafy vegetables, a few percent typically show inhibitory activity against tested pathogens.[15,16] With the design used for this study, it was not possible to identify orders with antagonistic effects to inoculated pathogens. In addition, if *Pseudomonas* strains with antagonistic effect were present in our samples, the abundance was likely to be too low to have any significant effect on the inoculated pathogens. However, Pseudomonadales was negatively correlated with viable counts of *E. coli* O157:H7 *gfp+*, which may indicate that these may have any type of antagonistic interaction. *Pseudomonas* with antagonistic effects have previously been suggested to provide new targets for biocontrol of potential human pathogens in RTE leafy vegetables [8] since they are well adapted to the phyllosphere environment and appear to persist during post-harvest operations.[15]

This study also indicated that Enterobacteriales (represented by family *Enterobacteriaceae* including *Erwinia* and unclassified *Enterobacteriaceae*) increased at both 8 and 15°C and that the largest increase was recorded at 15°C, representing temperature abuse. Members of the *Enterobacteriaceae* are often among the most abundant members of the phyllosphere community in studies of vegetable salads, particularly at temperature abuse.[8,14] Since many human pathogens belong to *Enterobacteriaceae*, this highlights the increased risk of foodborne disease when leafy vegetables with fecal contamination have been subjected to temperature abuse.

Based on the PLS model, there were positive correlations between viable counts of inoculated strains on day 7 and the parallel abundance of the orders Bacillales, Lactobacillales, and Enterobacteriales. This was overall also supported by Spearman rank sum correlations. Some of these correlations were not surprising, since *L. monocytogenes* is affiliated to Bacillales while pathogenic *Y. enterocolitica* and *E. coli* O157:H7 *gfp*+ are affiliated to Enterobacteriales. Reasons for these correlations may be that inoculants and bacteria included in these orders are stimulated by the same conditions or possibly that they facilitate each other’s growth by some unknown synergistic mechanisms.

To avoid biases from uneven sampling efforts, all samples were normalized, i.e. randomly subsampled to 1000 sequences in our microbiota analyses. To avoid biases from sequencing errors and singletons, OTUs with less than 10 reads were discarded from each sample. This sequencing depth theoretically enables detection of populations making up more than 1% of the total community. Our results indicate that in salads where background concentrations of indigenous microorganisms are likely to be high, pathogens present in viable numbers of 10^7^ CFU g^−1^ will hence not be robustly identified in OTU tables from the Illumina sequencing performed at the sequencing depth used in this study. Nevertheless, BLAST against reference sequences from *Yersinia*, *Escherichia/Shigella*, and *Listeria* resulted in OTUs with 96 to nearly 100% identity, indicating a potential for using next-generation sequencing analyses of 16S rRNA amplicons for pathogen monitoring. Identification of inoculated strains was only possible for samples where these strains were present in high viable counts, suggesting that greater sequencing efforts would have been valuable. The inoculated *L. monocytogenes* strain was identified amongst OTUs when viable concentrations of this pathogen was at the highest level; 10^9^ CFU g^−1^. Since total aerobic counts (based on culture methods) were approximately 10^10^ CFU g^−1^ in corresponding samples,[21] *L. monocytogenes* represented 10% of total viable counts. Based on the molecular method used in this study, the OTU that represented inoculated *L. monocytogenes* contributed merely 2.2% of the total reads for these specific samples. Consequently this indicates that the molecular method detected 4–5 times more bacteria present in the background microbiota of the sample compared to total viable counts detected by the culturing method. The difference is likely due to accumulation of damaged or dead microbial cells during the incubation. In this study, the OTU that appeared to represent the inoculated *E. coli* O157:H7 *gfp+* was identified only after studying the OTU list prior to the subsampling-based normalization and it was only observed in samples with the highest viable counts of *E. coli* O157:H7 *gfp+* (approximately 10^7^ CFU g^−1^). These were samples of mixed-ingredient salad stored at 15°C for seven days, where the inoculated strain accounted for 0.1% of total aerobic viable counts.[21] Based on the molecular method used in this study, the OTU that represented inoculated *E. coli* O157:H7 *gfp*+ contributed 0.2% of the total community in these specific samples. The inoculated pathogenic *Y. enterocolitica* was not identified with the DNA-based method despite being present at the same level as *E. coli* O157:H7 *gfp*+ in some samples, with viable counts of 10^7^ CFU g^−^^1^.[21] The viable counts of *L. monocytogenes* and pathogenic *Y. enterocolitica* that were observed in the mixed-ingredient salad stored at temperature abuse are levels that may cause human disease following consumption.[34–36] Since *E. coli* O157:H7 may cause disease at a very low level (<100 cells),[37] the inoculated salads presented a risk already before storage. Hence, to increase the chance of detecting specific bacterial populations (e.g. pathogens) that may account for a minor proportion of the total bacterial community while still representing a potential health hazard, both higher sequencing depth and identification at lower taxonomic level, i.e. at species level, will be necessary.

We used homogenization to retrieve microbiota both from leaf surface and inside of leaves, since this combined microbiome is what consumers are exposed to. Homogenizing and sonication, i.e. pulsifying to detach bacteria tightly adhered to leaf surface,[30] have been shown to recover similar quantities and diversity of bacteria.[38] However, microbiota from leafy vegetables may also be retrieved in other ways, such as from wash water,[28] illustrating that there is yet no standard approach for analyzing the microbiota of leafy vegetables. Another example is that different DNA extraction methods or primers may be used in different studies, which may have a major effect on the outcome of the analysis and should be considered when comparing studies.[39] Standardized methodologies for analyzing the microbiota of leafy vegetables would be helpful to reduce biases and enable comparative analyses across studies.

## Conclusions

In was clear that the composition of bacterial communities changed during storage, but Pseudomonadales remained the most abundant order across all samples. Positive correlations were identified between viable counts of inoculated strains and abundances of Lactobacillales, Enterobacteriales, and Bacillales, pointing to positive interactions or similar environmental driver variables that may make it feasible to use such bacterial lineages as indicators of microbial health hazards in leafy vegetables. The viable counts of inoculated strains only represented a small fraction of the total viable counts in the salads, thus they were in general not detected among OTUs.

## Supplementary Material

Supplemental Data
