# Supplementary material for: Emerging microbiota during cold storage and temperature abuse of ready-to-eat salad​
Source: Infect Ecol Epidemiol. 2017 Jun 6;7(1):1328963. doi: 10.1080/20008686.2017.1328963 (PMC5475331; doi:10.1080/20008686.2017.1328963)
Supplement: Supplemental Data [file ziee_a_1328963_sm2610.docx]

**Supplementary table 1**. Relative abundance (± standard error) of bacterial phyla associated with control samples of baby spinach and mixed-ingredient salad (baby spinach mixed with chicken meat) at different storage temperatures (8 and 15 °C).

|  | Proteobacteria | Bacteroidetes | Firmicutes | Actinobacteria |
| --- | --- | --- | --- | --- |
| Baby spinach | |  |  |  |
| Day 0 | 0.628 ± 0.079 | 0.347 ± 0.069 | 0.007 ± 0.003 | 0.017 ± 0.010 |
| Day 7, 8 °C | 0.749 ± 0.126 | 0.232 ± 0.131 | 0.011 ± 0.010 | 0.005 ± 0.002 |
| Day 7, 15 °C | 0.733 ± 0.112 | 0.249 ± 0.120 | 0.010 ± 0.007 | 0.007 ± 0.002 |
| Mixed-ingredient salad | |  |  |  |
| Day 0 | 0.670 ± 0.097 | 0.287 ± 0.058 | 0.003 ± 0.002 | 0.037 ± 0.034 |
| Day 7, 8 °C | 0.855 ± 0.075 | 0.108 ± 0.090 | 0.030 ± 0.019 | 0.007 ± 0.004 |
| Day 7, 15 °C | 0.783 ± 0.085 | 0.168 ± 0.100 | 0.035 ± 0.018 | 0.013 ± 0.011 |

**Supplementary table 2.** Number of paired end reads before and after processing.

| Sample number | Paired reads | Absolute number of reads | Reads lost during sequence analysis |
| --- | --- | --- | --- |
| 1 | 15826 | 2302 | 13524 |
| 2 | 10715 | 2214 | 8501 |
| 3 | 8942 | 7220 | 1722 |
| 4 | 3 | 2 | 1 |
| 5 | 28354 | 23028 | 5326 |
| 6 | 28947 | 23241 | 5706 |
| 7 | 9735 | 8126 | 1609 |
| 8 | 19114 | 11752 | 7362 |
| 9 | 15430 | 11808 | 3622 |
| 10 | 18279 | 12366 | 5913 |
| 11 | 29004 | 24220 | 4784 |
| 12 | 17910 | 9945 | 7965 |
| 13 | 19813 | 15213 | 4600 |
| 14 | 8692 | 5420 | 3272 |
| 15 | 11828 | 9770 | 2058 |
| 16 | 14214 | 6419 | 7795 |
| 17 | 7953 | 6109 | 1844 |
| 18 | 9359 | 6535 | 2824 |
| 19 | 11700 | 990 | 10710 |
| 20 | 23931 | 209 | 23722 |
| 21 | 133 | 105 | 28 |
| 22 | 13952 | 9976 | 3976 |
| 23 | 6141 | 5379 | 762 |
| 24 | 5677 | 4474 | 1203 |
| 25 | 6772 | 5717 | 1055 |
| 26 | 17164 | 6049 | 11115 |
| 27 | 14416 | 11538 | 2878 |
| 28 | 11906 | 9496 | 2410 |
| 29 | 18290 | 16770 | 1520 |
| 30 | 25202 | 12392 | 12810 |
| 31 | 24451 | 19287 | 5164 |
| 32 | 16077 | 12623 | 3454 |
| 33 | 30364 | 22865 | 7499 |
| 34 | 14347 | 6817 | 7530 |
| 35 | 21375 | 16615 | 4760 |
| 36 | 9581 | 5439 | 4142 |
| 37 | 52897 | 15874 | 37023 |
| 38 | 2371 | 1342 | 1029 |
| 39 | 8950 | 7793 | 1157 |
| 40 | 15223 | 5258 | 9965 |
| 41 | 11725 | 9986 | 1739 |
| 42 | 31400 | 24916 | 6484 |
| 43 | 11592 | 7745 | 3847 |
| 44 | 6983 | 4345 | 2638 |
| 45 | 2697 | 2304 | 393 |
| 46 | 6514 | 5046 | 1468 |
| 47 | 18265 | 15923 | 2342 |
| 48 | 5140 | 4226 | 914 |
| 49 | 59383 | 49896 | 9487 |
| 50 | 4819 | 3736 | 1083 |
| 51 | 9548 | 8257 | 1291 |
| 52 | 3652 | 2784 | 868 |
| 53 | 7704 | 6596 | 1108 |
| 54 | 7800 | 6127 | 1673 |

**Supplementary table 3.** 16S sequences for the inoculated strains.

| **Inoculated strain** | **16S sequence** |
| --- | --- |
| *Listeria monocytogenes* | TTTCGGATCGTAAAGTACTGTTGTTAGAGAAGAACAAGGATAAGAGTAACTGCTTGTCCCTTGACGGTATCTAACCAGAAAGCCACGGCTAACTACGTGCCAGCAGCCGCGGTAATACGTAGGTGGCAAGCGTTGTCCGGATTTATTGGGCGTAAAGCGCGCGCAGGCGGTCTTTTAAGTCTGATGTGAAAGCCCCCGGCTTAACCGGGGAGGGTCATTGGAAACTGGAAGACTGGAGTGCAGAAGAGGAGAGTGGAATTCCACGTGTAGCGGTGAAATGCGTAGATATGTGGAGGAACACCAGTGGCGAAGGCGACTCTCTGGTCTGTAACTGACGCTGAGGCGCGAAAGCGTGGGGAGCAAACAGGATTAGATACCCCNGTAGTCA |
| *Escherichia coli* | CTTCGGGNTGTAAAGTACTTTCAGCGGGGAGGAAGGGAGTAAAGTTAATACCTTTGCTCATTGACGTTACCCGCANAAGAAGCACCGGCTAACTCCGTGCCAGNAGCCGCGGTAATACGGAGGGTGCAAGCGTTAATCGGAATTACTGGGCGTAAAGCGCACGCAGGCGGTTTGTTAAGTCAGATGTGAAATCCCCGGGCTCAACCTGGGAACTGCATCTGATACTGGCAAGCTTGAGTCTCGTAGAGGGGGGTAGAATTCCAGGTGTAGCGGTGAAATGCGTAGAGATCTGGAGGAATACCGGTGGCGAAGGCGGCCCCCTGGACGAAGACTGACGCTCAGGTGCGAAAGCGTGGGGAGCAAACAGGATTAGATACCCC |
| *Yersinia enterocolitica* | CTTCGGGTTGTAAAGCACTTTCAGCGAGGAGGAAGGCATAAAGGTTAATAACCTTTGTGATTGACGTTACTCGCAGAAGAAGCACCGGCTAACTCCGTGCCAGCAGCCGCGGTAATACGGAGGGTGCAAGCGTTAATCGGAATTACTGGGCGTAAAGCGCACGCAGGCGGTTTGTTAAGTCAGATGTGAAATCCCCGCGCTTAACGTGGGAACTGCATTTGAAACTGGCAAGCTAGAGTCTTGTAGAGGGGGGTAGAATTCCAGGTGTAGCGGTGAAATGCGTAGAGATCTGGAGGAATACCGGTGGCGAAGGCGGCCCCCTGGACAAAGACTGACGCTCAGGTGCGAAAGCGTGGGGAGCAAACAGGATTAGATACCCCNGTAGTCA |
